# Vaccination: short- to long-term benefits from investment

**DOI:** 10.3402/jmahp.v3.27279

**Published:** 2015-08-12

**Authors:** Stuart Carroll, Amós José García Rojas, Anna H. Glenngård, Carmen Marin

**Affiliations:** 1 Sanofi Pasteur MSD, Maidenhead, UK; 2 Spanish Association for Vaccinology, Spain; 3 Lund University School of Economics and Management, Lund, Sweden; 4 Sanofi Pasteur MSD, Madrid, Spain

**Keywords:** Vaccination, investment, short-term, benefits, costs, budget

## Abstract

In the context of current economic difficulties across Europe, accurate budgeting and resource allocation have become increasingly important. Vaccination programmes can respond to the needs of governments to budget with confidence. It may be more reliable and accurate to forecast budget and resource allocation for a vaccination programme than for unpredictable seasonal disease peaks of infections such as rotavirus gastroenteritis, influenza, and pneumonia. In addition, prevention through vaccination involves low levels of investment relative to the substantial benefits that may be obtained. In France, total lifelong vaccination costs, per fully compliant individual, ranged from €865 to €3,313, covering 12 to 16 diseases, which is comparable to, or lower than, costs of other preventive measures. In addition, effectively implemented vaccination programmes have the potential to generate substantial savings both in the short and in the long term. For example, vaccination programmes for rotavirus, meningitis C, human papillomavirus, influenza, and pneumonia have all been shown to significantly reduce the disease burden, and thus the associated costs, in the first years following vaccination implementation. These programmes demonstrate the potential for health authorities to obtain early, and often substantial, return on investment.

In Europe, a small percentage of national health care budgets is allocated to prevention (3% in average), and only a small part of it is allocated to vaccination. More than three-quarters of OECD countries reported a cut in real-term spending on prevention programmes in 2011 over 2010, and half spent less than in 2008 (1). Indeed, preventive programmes are most vulnerable to budget cuts and restrictions as their benefits may not be always immediately identifiable whilst cuts often focus on short-term financial results. However, any short-term benefits to budgets are likely to be greatly outweighed by the long-term impact on health and spending (1, 2).

Vaccination is often considered a long-term investment only, overlooking the potential short- and medium-term benefits. However, when implemented efficiently, vaccination programmes can also provide early and substantial returns on investment.

In this paper, we will examine the role and advantages of vaccination programmes in health care budget planning, the cost of lifelong vaccination strategies compared with other preventive measures, and the short-term benefits that have been reported for some recently introduced vaccines.

## Vaccination programmes can contribute to accurate health care budgeting

Scarce resources for health make accurate health care budgeting and resource planning essential for achieving important health systems goals. This is essential for achieving value for money and ensuring that budget holders can plan and manage public health expenditures with reasonable precision. It is also fundamental for the sustainability of health care systems that need to respond to the imperatives of continuous reform. Such principles are embedded in European and national guidelines for economic evaluation.

Vaccination programmes can respond to the imperative of helping governments budget with confidence. It is possible for health care systems to accurately forecast the upfront costs of vaccination programmes, or at least to estimate the maximum budget assuming 100% uptake, whereas it is more difficult to precisely forecast and budget for the variable costs of treating vaccine-preventable diseases. Indeed, while disease dynamics and future epidemiology are equally uncertain when modelling drugs and vaccines benefits, predicting treatment costs may be subject to considerable uncertainty since several behavioural and environmental parameters need to be taken into consideration (3).

If we take the example of rotavirus vaccination, from a budget forecast point of view, it is easier to estimate accurately the costs for a vaccination programme than to estimate the variable (and uncertain) costs of treating preventable rotavirus gastroenteritis cases every year, particularly since the incidence and severity of disease vary between seasons. Moreover, these estimated treatment costs may not reflect the full financial burden to health care systems and society as productivity and opportunity costs are very difficult to evaluate. This is also true for other diseases that are associated with annual peaks or seasonality, such as influenza and pneumonia. In conclusion, compared with treatment-focused interventions that have deferred costs distributed over several years according to the disease epidemiology, prevention-focused strategies have budgetable short-term costs which are concentrated in time. This better predictability of expenses achieved through vaccination could bring an additional ‘safety’ value for budget holders.

## Total direct costs of vaccination throughout life are comparable to or lower than those for other preventive measures

Within health care expenditures, prevention spendings represent a small percentage (<3%) of total expenditures in Europe (1). In France, total expenditure on vaccines represented an estimated 0.3% of total health care expenditure in 2013 (4).

**Fig. 1:**
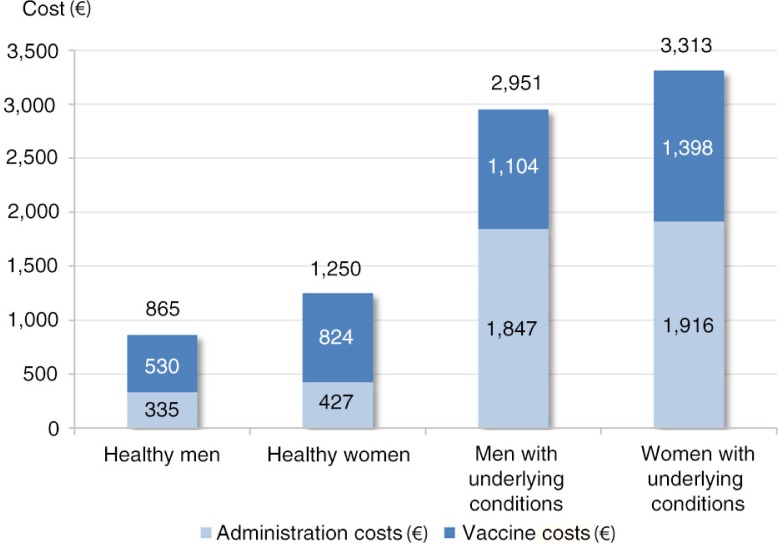
Lifelong vaccination costs per fully compliant individual in France (5).

A recent study estimated that the lifelong costs per individual vaccinated in full compliance with national recommendations in France ranged from €524 (men without underlying conditions) to €1,379 (women with underlying conditions) in 2014, covering 12 to 16 diseases, respectively, and from €865 to €3,313 when including administration costs (Fig. 1) (5). It was estimated that lifelong vaccine costs were 4 to 10 times lower than costs of statins in the treatment of hypertension; 7 to 14 times lower than the costs for DPP-4 inhibitors in the treatment of type 2 diabetes; and 6 to 13 times lower than costs for antithrombotic medication in the prevention of recurrent stroke. Hence, prevention through vaccination requires low levels of investment relative to the substantial benefits that can be obtained.

## Short-term value and rapid return on investment

Policy makers are interested in costs and effectiveness in both the short and the long term when deciding which health care technologies they want to invest in. The impact and potential savings in disease treatment costs due to vaccination programmes often occur many years after the implementation of the programme, which makes cost-effectiveness studies of vaccination programmes subject to some uncertainty before real-world data can confirm their predictions. In addition, the discounting of health outcomes imposed by cost-effectiveness guidelines devalues the long-term benefits of prevention programmes such as vaccination, compared with short-term interventions (6).

**Fig. 2:**
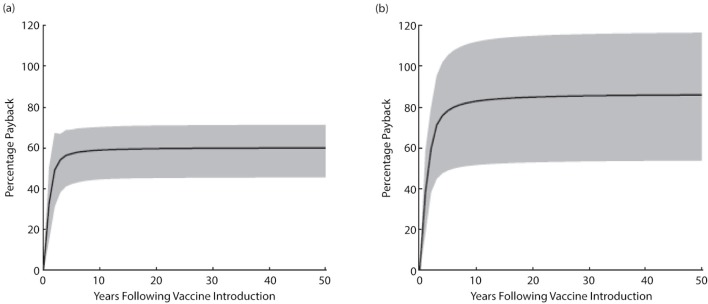
Budget impact analysis showing the cumulative mean annual percentage payback predicted for introduction of rotavirus vaccination in England and Wales (7). (a) Immediate vaccine immunity waning after vaccination and (b) delayed vaccine immunity waning after vaccination. Price for a full-course regimen was assumed to be £60.

However, there are examples of vaccination programmes that generate visible short-term savings. One such example is the recent implementation of rotavirus vaccination in several European countries. Rotavirus is highly contagious and resistant to most soaps and disinfectants. Symptoms often include vomiting and fever as well as diarrhoea which, in severe cases, can result in life-threatening dehydration. A UK cost-effectiveness analysis showed that introducing a rotavirus vaccination programme could pay back between 58 and 96% of the outlay costs for vaccination within the first four years of the programme (Fig. 2) (7). Another study in Italy showed that, as early as the second year after programme introduction, the costs of the vaccine would be more than offset by savings from prevented cases of rotavirus gastroenteritis and reduced number of hospitalisations (8). Savings amounted to €34,440 over 5 years, which is equivalent to €4.64 per child from the perspective of the national health service. The estimated economic impact of introducing the vaccine would have been even higher if costs for absenteeism and loss of productivity had been taken into consideration. In countries where rotavirus vaccination programmes have been implemented, a clear clinical benefit has already been reported, confirming predictions from cost-effectiveness models. The most recent example comes from the newly introduced national infant rotavirus vaccination programme in the United Kingdom (implemented in summer 2013). Data on the impact of the immunisation programme in England after its first full year showed a 71% reduction in laboratory-confirmed cases (9). Significant reductions were also seen for the number of GP-reported cases and those from Accident & Emergency departments.

**Fig. 3:**
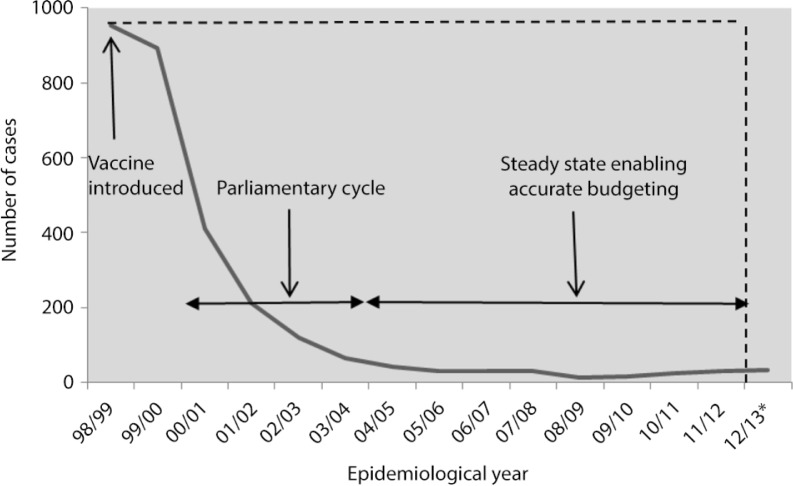
Annual number of meningitis C cases from 1998/1999 to 2009/2010* (10). *Provisional data.

Another example of short-term savings from vaccination comes from the experience in the United Kingdom with their meningitis C vaccination programme (Fig. 3) (10). The United Kingdom was the first country to introduce meningococcal serogroup C conjugate vaccination in November 1999. Within 5 and 10 years of vaccine introduction, the number of reported cases declined by 93 and 99%, respectively, compared to baseline. After 10 years, it was estimated that the cumulative savings from the approximately 9,000 cases avoided since the start of the vaccination was £75 million (10, 11). Although this could be an overestimation as the cost per case was calculated based on resources required for treating those aged between 0 and 17 years (who incur higher long-term treatment costs in the event of complications), substantial savings are confirmed.

Quadrivalent human papillomavirus (4HPV) vaccination programmes provide another example of short-term value and return on investment. Prior to introduction, a model applied to Norway estimated that 90% of all costs avoided during the first 5 years of a quadrivalent HPV vaccination programme in 12- to 24-year-old girls would be due to avoided HPV6/11 diseases (i.e., genital warts, or GWs) (12). The findings from this model were also confirmed by real-world data in settings where high coverage rates were achieved. For example, between 2007 and 2009, Australia introduced HPV quadrivalent vaccination for all females aged to 12–26 years. There was an uptake of about 70% for three doses in the school-age cohort (13). By the end of 2009, an Australian nationwide surveillance programme using data from eight sexual health centres reported a 59% reduction in the number of new cases of GWs in females aged 12–26 years (13). By mid-2011, 4 years after the start of the vaccination programme, another Australian study showed that large reductions in new GW episodes occurred in women aged 21 years (92.6%) and 21–30 years (72.6%) compared with the pre-vaccination period (14). This study also reported an important decline in GWs in heterosexual men, which is most likely attributable to an indirect ‘herd protection’ as they were not part of the HPV vaccine target population.

These examples of vaccination short-term benefits confirm that, in addition to long-term impact on population health, vaccination can also provide rapid and substantial returns on investment if implemented efficiently.

## Conclusions

Vaccination plays an important role in public health strategies conferring predictability and confidence in budgeting. Disease prevention through vaccination requires low levels of investment relative to the substantial short-term and long-term benefits that can be obtained. Although vaccines provide a range of long-term benefits, it is important that policy makers consider also the short-term benefits when taking decisions about the introduction of vaccination. There is, therefore, a strong case for consideration of return on investment analyses, which may better reflect the short-term benefits of vaccination, as an addition to cost-effectiveness analyses which focus on a longer time horizon and may devalue the long-term benefits of prevention programmes compared to the short-term benefits of curative programmes. This is essential for resource allocation decisions concerning public health budgets and wider health care interventions.
